# C4b Binding Protein Acts as an Innate Immune Effector Against Influenza A Virus

**DOI:** 10.3389/fimmu.2020.585361

**Published:** 2021-01-08

**Authors:** Praveen M. Varghese, Valarmathy Murugaiah, Nazar Beirag, Nigel Temperton, Haseeb A. Khan, Salman H. Alrokayan, Mohammed N. Al-Ahdal, Beatrice Nal, Futwan A. Al-Mohanna, Robert B. Sim, Uday Kishore

**Affiliations:** ^1^ Biosciences, College of Health and Life Sciences, Brunel University London, Uxbridge, United Kingdom; ^2^ School of Biosciences and Technology, Vellore Institute of Technology, Vellore, India; ^3^ Viral Pseudotype Unit, Medway School of Pharmacy, University of Kent and Greenwich, Kent, United Kingdom; ^4^ Department of Biochemistry, College of Science, King Saud University, Riyadh, Saudi Arabia; ^5^ Department of Cell Biology, King Faisal Specialist Hospital and Research Centre, Riyadh, Saudi Arabia; ^6^ Department of Infection and Immunity, King Faisal Specialist Hospital and Research Center, Riyadh, Saudi Arabia; ^7^ Department of Biochemistry, University of Oxford, Oxford, United Kingdom

**Keywords:** complement, C4BP, influenza A virus, inflammation, pseudo-typed lentiviral particles

## Abstract

C4b Binding Protein (C4BP) is a major fluid phase inhibitor of the classical and lectin pathways of the complement system. Complement inhibition is achieved by binding to and restricting the role of activated complement component C4b. C4BP functions as a co-factor for factor I in proteolytic inactivation of both soluble and cell surface-bound C4b, thus restricting the formation of the C3-convertase, C4b2a. C4BP also accelerates the natural decay/dissociation of the C3 convertase. This makes C4BP a prime target for exploitation by pathogens to escape complement attack, as seen in Streptococcus *pyogenes* or Flavivirus. Here, we examined whether C4BP can act on its own in a complement independent manner, against pathogens. C4BP bound H1N1 and H3N2 subtypes of Influenza A Virus (IAV) most likely *via* multiple sites in Complement Control Protein (CCP) 1-2, 4-5, and 7-8 domains of its α-chain. In addition, C4BP CCP1-2 bound H3N2 better than H1N1. C4BP bound three IAV envelope proteins: Haemagglutinin (~70 kDa), Neuraminidase (~55 kDa), and Matrix protein 1 (~25kDa). C4BP suppressed H1N1 subtype infection into the lung epithelial cell line, A549, while it promoted infection by H3N2 subtype. C4BP restricted viral entry for H1N1 but had the opposite effect on H3N2, as evident from experiments using pseudo-typed viral particles. C4BP downregulated mRNA levels of pro-inflammatory IFN-α, IL-12, and NFκB in the case of H1N1, while it promoted a pro-inflammatory immune response by upregulating IFN- α, TNF-α, RANTES, and IL-6 in the case of H3N2. We conclude that C4BP differentially modulates the efficacy of IAV entry, and hence, replication in a target cell in a strain-dependent manner, and acts as an entry inhibitor for H1N1. Thus, CCP containing complement proteins such as factor H and C4BP may have additional defense roles against IAV that do not rely on the regulation of complement activation.

## Introduction

The complement system plays a crucial role in our early response against invading pathogens, including viruses. The complement system is activated *via* three pathways—Classical, Alternative, and Lectin. The classical pathway is activated through the binding of C1q to IgG or IgM-containing immune complexes or other non-immunoglobulin targets. Non-self-carbohydrate targets are recognized by Mannose Binding Lectin (MBL) or ficolins triggering the lectin pathway. The activation of classical or lectin pathways leads to the cleavage of C4 and C2, yielding C3 convertase (C4b2a), which then cleaves C3 to form C3b. For the alternative pathway, C3 is spontaneously hydrolyzed to a C3b-like form [C3(H_2_O)] due to the hydrolysis of the internal thioester bond. The C3(H_2_O) binds to Factor B, which enables Factor D to cleave Factor B to Bb. This then forms C3(H_2_O)Bb, which is homologous to the C3 convertase, C4b2a. C3(H_2_O)Bb then cleaves C3 to C3b, and C3b can bind covalently to target surfaces, on which it forms more convertase, C3bBb. The binding of the C3b to C4b2a or C3bBb converts them into classical or alternative pathway C5 Convertases, respectively. The subsequent cleavage of C5 by the C5 convertases initiates the formation of the Membrane Attack Complex (C5b-C9). This complex binds to the microbial surface and can cause lysis of lipid bilayer membranes (1).

The complement system is kept in check by various regulatory proteins to prevent runaway reactions leading to unnecessary inflammation and death of healthy cells. One such humoral regulator is C4b Binding Protein (C4BP). C4BP, like other complement regulators Factor H, CR1, CD46, CD55, and the Factor H-related family, is encoded by a gene present in the long arm of chromosome 1 and is synthesized and secreted mainly by hepatocytes (2). The normal range of C4BP in human plasma is estimated to be approximately 150–300 µg/ml (3). C4BP regulates complement activation by controlling C4b mediated reactions (4). C4BP, a 570 kDa glycoprotein, functions as a primary fluid phase regulator of the classical and lectin pathways (5). It consists of seven identical 70 kDa α-chains and a 45 kDa β-chain that are linked at their carboxy-terminal ends by short amphipathic helices further stabilized by disulfide bonds (6). The α- chains and β-chain contain eight and three Complement Control Protein (CCP) domains, each of ~60 amino acids, respectively. The polymerization of the polypeptide chains of C4BP occurs *via* a short C-terminal region, downstream of CCP8 (7). Each molecule of C4BP can bind only four molecules of C4b due to steric hindrance, although each of the seven α- chains has a C4b binding site (8). The binding site for C4b has been localized to CCP1-3, especially within a cluster of positively charged residues at the interface of CCP1 and CCP2 (9, 10). C4BP has been shown to inhibit the formation of C3 and C5 convertases, accelerate the decay of the convertases, and act as a co-factor for Factor I which cleaves and thereby inactivates fluid phase and cell-bound C4b (9–12). The co-factor activity of the protein has been linked to CCP1-4 (13).

Consistent with its role as an immunomodulator, C4BP also engages with pathogens. Flaviviruses limit complement activation by binding C4BP through their NS1 protein: the bound C4BP inactivates soluble or membrane-bound C4b (5). *Streptococcus pyogenes* uses its Protein H to recruit C4BP in order to facilitate the invasion of endothelial cells and reduce the chances of its opsonization by bound C3b, or attack by the MAC (14). C4BP probably is also recruited by other pathogens to limit C3 opsonization, MAC mediated lysis, and immune system activation by C3a/C5a (15).

C4BP has been reported to have properties that are independent of its involvement in complement regulation. For example, C4BP is known to facilitate the uptake of adenoviruses by hepatocytes *via* its interaction with heparin-sulfate proteoglycans found on cell surfaces (16). C4BP induces proliferation and activation of B lymphocytes by binding with CD40 (17). Interaction between CD40, CD154, and C4BP inhibits apoptosis in epithelial cells. C4BP has also been implicated in inhibiting DNA release from necrotic cells in conjunction with Protein S by binding to phosphatidylserine (18–20). C4BP also have an association with the clotting system as the β-chain binds the vitamin K-dependent anticoagulant protein S (21).

In this study, we investigated potential complement-independent defense functions of C4BP against Influenza A Virus (IAV). Influenza virus causes an upper respiratory tract infection affecting the nose, throat, bronchi, and sometimes the lungs. Of the three genera of Influenza virus that belong to the Orthomyxoviridae family, IAV is known to cause pandemics. IAV causes Influenza in birds and a few species of mammals, including humans and pigs. Its wide host range has led to pandemics such as the 1918 Spanish flu, which caused 20–50 million deaths (22). It has several subtypes based on variants of two proteins on the surface of the viral envelope. These proteins are Hemagglutinin (HA), which causes RBCs to agglutinate, and Neuraminidase (NA), which breaks the glycoside bonds of neuraminic acid (23). Currently, there are 18 known variations of HA, and 11 known variations of NA, theoretically allowing 198 different combinations (24, 25). Some of the well-known combinations are H1N1 that caused the 1918 Spanish flu, 1977 Russian flu, and 2009 flu Pandemic, H3N2 that caused the 1968 Hong Kong flu and H2N2 that caused 1957 Asian flu (26).

Human infection by IAV is achieved through oral or nasal cavities, where the viral HA binds sialic acids in order to attach to lung epithelial cells (27). The HA protein of the human-infecting IAV has increased specificity for α-2,6-linked sialic acid (28). Sialic acid, a nine-carbon acidic monosaccharide, is commonly found with α-2,6-linkages and a few α-2,3-linkages on human tracheal epithelial cells (29). Once bound to the cell surface, the viral particle undergoes receptor-mediated endocytosis. We show here that IAV interacts with the α chain of C4BP mainly *via* CCP domains 4–5, CCP domains 7–8, and to a lesser extent CCP domains 1–2. C4BP acts as an entry inhibitor for H1N1 subtype when challenged against A549 (lung epithelial) cells and suppresses the pro-inflammatory cytokine storm induced by IAV.

## Materials and Methods

### Purification of Native C4BP

C4BP was isolated from human plasma as described previously (30). Briefly, C4BP was purified from neutral euglobulin precipitate by affinity chromatography using C4c-Sepharose, equilibrated in 5 mM EDTA/25 mM potassium phosphate buffer, pH 7.0. The column was washed in the same buffer and then bound protein was eluted by a linear gradient of NaCl from 0 to 2 M; C4BP was eluted at 0.8 M NaCl. The peak fractions were run on 12% w/v acrylamide SDS-PAGE to assess purity and adjusted to 1 mg/ml concentration. The immunoreactivity of this plasma purified C4BP was assessed *via* western blotting.

### Expression and Purification of Recombinant Complement Control Protein Deletants of C4BP α-Chain

Recombinant α-chain of C4BP (rC4BP) and C4BP α-chain CCP deletants (**Table 1**) were provided by Prof. Anna Blom, Lund University, Malmo, Sweden. Briefly, CCP deletant constructs were expressed in Human Embryonic Kidney (HEK) 293 cells (ATCC number 1573-CRL). The transfected cells were selected using neomycin analogue G418 (400 μg/ml), and colonies expressing the highest amount of CCP deletants were assessed by immunoblotting. The medium collected from transfected cells was subjected to the purification of CCP deletants using mAb 104 affinity columns (directed against CCP1) or mAb 67 (directed against CCP4) coupled to Affi-Gel 10 (2.6 × 12 cm column; Bio-Rad) (10). The recombinant CCP deletants were eluted using 3M guanidinium chloride, and the purified fractions were subjected to SDS-PAGE with 12% w/v acrylamide/bisacrylamide (10).

**Table 1:** C4BP deletants used in this study.

Mutants	Deleted regions within the α-chain of C4BP
ΔCCP1	Asn^1^–Tyr^62^
ΔCCP2	Lys^63^–Ile^124^
ΔCCP3	Val^125^–Lys^188^
ΔCCP4	Lys^188^–Asn^249^
ΔCCP5	Asn^249^–Ala^314^
ΔCCP6	Leu^315^–Gly^375^
ΔCCP7	Asp^376^–Lys^433^
ΔCCP8	Cys^436^–Trp^492^

### Preparation of Viral Stocks and Titration

A/England/2009 (H1N1) was a gift from Wendy Barclay, Department of Infectious Disease, Imperial College London, and the A/HK/99 (H3N2) was a gift from Leo Poon, Division of Public Health Laboratory Sciences, University of Hong Kong. Madin Darby Canine Kidney (MDCK) (ATCC^®^ CCL-34™) cells, grown to 70% confluence in a 175 cm^2^ flask (Fisher Scientific) in growth medium (DMEMF/12 with GlutaMAX™ Supplement (Gibco), + 10% v/v Heat Inactivated Fetal Bovine Serum (FBS) (Gibco) + 1% v/v Penicillin-Streptomycin (PS) (Gibco) at 37°C under 5% v/v CO_2_, were washed with PBS twice to remove any residual serum-complemented DMEM media. H1N1 and H3N2 IAV subtypes were added to the MDCK cells diluted in serum-free DMEM (Gibco) and allowed to incubate for 1 h at 37°C. The medium was removed, and cells were washed with Phosphate-buffered saline (PBS) (0.01M sodium phosphate, 0.0027M KCl, and 0.137M NaCl, pH 7.4 at 25°C) (Fisher Bioreagents) to remove any unbound viruses. The cells were then incubated for 3 days at 37°C in the infection medium [DMEM + 1% penicillin/streptomycin + 0.3% w/v bovine serum albumin (BSA) + 1 µg/ml of l-1-Tosylamide-2-phenylethyl chloromethyl ketone (TPCK)-Trypsin] (Sigma-Aldrich)]. TPCK treated Trypsin mediates the cleavage of HA0 into two subunits HA1 and HA2, which are required to make HA fusion competent. After the incubation, the medium was collected and centrifuged in sealed containers at 3,000 × g at 4°C for 15 min. The supernatant containing the viral particles was harvested, and the titer of the particles was determined *via* 50% Tissue culture Infective Dose (TCID_50_) Assay. Purified virus stocks were serially diluted in a 96-well plate up to 10^−7^-fold with ½ log_10_ increments. 1 × 10^5^ MDCK cells were added to each well and incubated for 3 days at 37°C under 5% v/v CO_2_. Uninfected MDCK cells were used as negative control. The titter of the virus was determined to be 1 × 10^6^ PFU/ml by TCID50 using the Reed–Muench method (31).

### Production of Pseudo-Typed Lentiviral Particles

Pseudo-typed Lentiviral Particles were produced as previously described by Murugaiah et al., 2020 (32). Briefly, HEK 293T cells were cultured in RPMI 1640 (Gibco), supplemented with 10% v/v heat inactivated Fetal Bovine Serum (FBS) (Gibco), 1% v/v Penicillin-Streptomycin (Gibco) at 37°C under 5% v/v CO_2_. Cells were co-transfected using 20 µg of the Envelope Protein-Coding plasmids that yield the viral envelope proteins [H1 and N1 or H3 and N2 or Vesicular Stomatitis Virus G protein (VSV-G)], an HIV-backbone containing a modified pro-viral HIV-1 genome with the *env* deleted and designed to express the firefly luciferase reporter and second or third-generation lentiviral vector packaging plasmid (**Table 2**). The culture supernatant was collected after 48 h (by centrifugation at 1,500 × g for 5 min). The transfected cells in the pellet were lysed using lysis buffer (50 mM Tris–HCl pH 7.5, 200 mM NaCl, 5 mM EDTA, 0.1% v/v Triton X-100) and were centrifuged at 1,500 × g for 5 min to remove cell debris. The total lysate and supernatant were filtered with a 22 µm filter. The pseudo-typed lentiviral particles were harvested by centrifuging the filtered cell lysate and the supernatant at 5,000 × g for 10 min at 4°C in a closed container. The lentiviral particles in the supernatant were then concentrated by ultracentrifugation at 25,000 × g for 3 h at 4°C (33). The pseudo-typed lentiviral particles produced were characterized by western blotting (32).

**Table 2:** Plasmids used for pseudo-typed lentivirus particle production.

	H1N1	H3N2	VSV-G
Envelope Protein-Coding Plasmid	pcDNA3.1-swineH1-flag (H1 from swine H1N1 A/California/04/09) (Codon optimized H1 (Genecust))	pcDNA-H3 [H3 from A/Denmark/70/03/(H3N2)] (Codon optimized H3 (Geneart))	pCMV-VSV-G (Addgene plasmid # 8454)
	pcDNA3.1-swine N1-flag (N1 from swine H1N1 A/California/04/09) (Codon optimized N1 (Genecust))	pI.18-N2 (N2 from human H3N2 A/Texas/50/2012)	
Backbone Plasmid	pHIV-Luciferase (Addgene plasmid # 21375)		
Packaging Plasmid	psPAX2 (Addgene plasmid # 12260)		

### ELISA to Detect C4BP-Virus Interaction

Microtiter wells of 96-well plates (Nunc # 4912) were coated (4°C, overnight) with the virus [100 µl of H1N1 or H3N2 subtypes (1,000 PFU/ml)] in carbonate-bicarbonate buffer, pH 9.6 (Sigma # c3041). Other wells were coated with 100 µl/well of C4BP, α-chain CCP mutants, at 5, 2.5, 1.25 µg/ml in the same buffer. In another experiment, 5 µg/ml of rC4BP was similarly coated. Unbound proteins or viruses were removed by washing the wells three times with PBST Buffer (PBS + 0.05% Tween 20) (Fisher Scientific). The wells were then blocked using 2% w/v BSA in PBS (Fisher Scientific) for 2 h at 37°C and washed three times using PBST. Decreasing concentrations of C4BP (5, 2.5, 1.25 µg/ml) were added to the wells in which the virus was coated to study its binding to immobilized viral particles. In parallel experiments, 1,000 PFU/ml of purified H1N1 and H3N2 were added to the protein-coated wells to study the binding of the virus to immobilized C4BP or rC4BP. The wells were incubated for 2 h at 37°C and then washed with PBST to remove any unbound viruses or protein. VSV-G pseudo-typed lentivirus was used as a negative control. The wells were probed using polyclonal rabbit anti-human C4BP (1 mg/ml of purified IgG; MRC Immunochemistry Unit, Oxford) to detect the virus-bound C4BP. Monoclonal Anti-Influenza Virus H1 HA, A/California/04/2009 (H1N1)pdm09, Clone 5C12 (produced *in vitro*) (BEI-Resources, NIAID, NIH, USA), Polyclonal Anti-Influenza Virus H3 HA, A/Hong Kong/1/1968 (H3N2) (antiserum, Goat, NR-3118) (BEI-Resources, NIAID, NIH, USA), and polyclonal anti-VSV-G (1 mg/ml of purified IgG; Imperial College London) were used to detect H1N1, H3N2 and pseudo-typed lentivirus particles, respectively that were bound to the immobilized C4BP. All antibodies were used at a dilution of 1:5,000 for 1 h at 37°C. Unbound antibodies were removed by washing three times using PBST. Anti-mouse IgG-Horseradish peroxidase (HRP) (Promega, #W4021), goat anti-rabbit IgG HRP (1:1,000; Promega # W4011), or Protein A-HRP (Invitrogen) were used as appropriate at 1:5,000 dilution as secondary antibodies (1 h at 37°C). 3,3′,5,5′-Tetramethylbenzidine (TMB) substrate set (Biolegend) was used as per the manufacturer’s instruction, and 1 M sulfuric acid (Fisher Chemical) was used to stop the reaction. The plate was read at 450 nm using an iMark™ microplate absorbance reader (BioRad).

### Western Blot

H1N1 and H3N2 (7.5 × 10^5^ PFU/ml), treated with NuPAGE LDS Sample Buffer (4X) (Invitrogen™) were subjected to 12% w/v reducing SDS-PAGE (120 min, 90 V). The proteins were then transferred to Polyvinylidene fluoride (PVDF) membranes (Millipore) by electroblotting (320 mA for 2 h) at 4°C. The membrane was then blocked using 5% w/v skimmed milk in PBS for 16 h at 4°C.

For far-western blot, the membrane was washed with PBS and incubated with 5 µg/ml of C4BP for 1 h at room temperature and another 1 h at 4°C on a rotary shaker. After washing with PBST, the PVDF membrane was probed using rabbit polyclonal anti-human C4BP antibody (1mg/ml) (1:1,000; MRC Immunochemistry Unit, Oxford) for 1 h at room temperature on a rotary shaker. Following three washes with PBST, the membrane was probed again with goat anti-rabbit IgG HRP conjugate (1:1,000; Promega #W4011) for 1 h; the blot was developed using 3,3′-diaminobenzidine (DAB) (Sigma) as a substrate.

For direct western blot for viral proteins, the PVDF membrane, containing electro-transferred viral proteins, was incubated with appropriate antibodies: these included monoclonal anti-IAV H1, polyclonal anti-IAV H3, Monoclonal anti-IAV Neuraminidase (NA), Clone NA2-1C1 (produced *in vitro*) (BEI-Resources, NIAID, NIH, USA), Polyclonal Anti-Influenza Virus N2 Neuraminidase (NA), A/shorebird/Delaware/127/1997 (H6N2) (antiserum, Goat) (BEI-Resources, NIAID, NIH, USA), Monoclonal Anti-Influenza A Virus Non-structural Protein 1 (NS1), Clone NS1-1A7 (produced *in vitro*) (BEI-Resources, NIAID, NIH, USA), or Monoclonal anti-Matrix protein 1 (M1) antibody (Abcam) as required for 1 h at room temperature on a rotary shaker. Following three washes with PBST, the membrane was probed with either anti-mouse HRP or Protein A-HRP conjugate, as appropriate for 1 h at room temperature on a rotary shaker. Following three washes with PBST, the blot was developed using DAB.

### A549 Cell Infection Assay

Human alveolar basal epithelial cells derived from an adenocarcinoma (A549) (ATCC^®^ CCL-185™) (5 × 10^5^) were cultured for 12 h in each well of a 12-well plate in growth medium. Post-attachment, the cells were serum starved by incubating them in serum-free DMEM for 24 h. H1N1 and H3N2 subtypes (MOI 1) were incubated with C4BP (10, 20, or 40 µg/ml) in PBS for 1 h and added to A549 cells in serum-free DMEM. After 1 h incubation at 37°C, the medium was removed, and cells were washed with PBS to remove any unbound IAV. Infection medium (DMEM + 1% v/v Penicillin-Streptomycin + 0.3% w/v BSA + 1 µg/ml of TPCK-Trypsin) was added to the cells and incubated for 2 or 6 h. The cells were then harvested by scraping with a sterile disposable cell scraper and centrifuging at 1,500 × g for 5 min. Total RNA was extracted using the GenElute™ Mammalian Total RNA Miniprep Kit (Sigma). Contaminating DNA was removed by treating the RNA samples with DNase I (Sigma) for 15 min. DNase I was inactivated by heating at 70°C for 10 min. The quantity and quality of the extracted RNA were assessed using a Nanodrop 2000 spectrophotometer (Thermo Fisher) *via* absorbance at 260 nm and 260:280 nm ratio. mRNA was converted to cDNA using TaqMan™ High Capacity RNA to cDNA Kit (Applied Biosystems™). The cDNA was stored at −20°C for future use. qRT-PCR was performed using a StepOnePlus System (Applied Biosystems™) at 95°C for 5 min, followed by 45 cycles of 95°C for 10 s, 60°C for 10 s, and 72°C for 10 s. The specificity of the assay was established by melting-curve analysis. The relative expression (RQ) was calculated by using C4BP untreated cells infected with respective viruses as the calibrator. The RQ value was calculated using the formula: RQ = 2^−ΔΔCt^. Primers were generated for specificity using the Primer-BLAST software (Basic Local Alignment Search Tool) (http://blast.ncbi.nlm.nih.gov/Blast.cgi) (**Table 3**).

**Table 3:** Forward and reverse primers used for qRT-PCR.

Target	Forward primer	Reverse primer
18S	5′-ATGGCCGTTC TTAGTTGGTG-3′	5′-CGCTGAGCCA GTCAGTGTAG-3′
IL-6	5′-GAAAGCAGCA AAGAGGCACT-3′	5′-TTTCACCAGG CAAGTCTCCT-3′
IL-12	5′-AACTTGCAGC TGAAGCCATT-3′	5′-GACCTGAACG CAGAATGTCA-3′
TNF-α	5′-AGCCCATGTT GTAGCAAACC-3′	5′-TGAGGTACAG GCCCTCTGAT-3′
M1	5′AAACATATGTCTGATAAC GAAGGAGAACAGTTCTT-3′	5′GCTGAATTCTACCT CATGGTCTTCTTGA-3′
RANTES	5′-GCGGGTACCAT GAAGATCTCTG-3′	5′-GGGTCAGAATC AAGAAACCCTC-3′
IFN-α	5′-TTT CTC CTG CC T GAA GGA CAG-3′	5′-GCT CAT GAT TTC TGC TCT GAC A-3′

### Luciferase Assay

MDCK cells were seeded in a 96-well plate (1 × 10^5^ cells per well) and were serum starved. The cells were infected for 24 h in the infection medium with 50 µl of the H1N1 and H3N2 pseudo-typed particles that were pre-incubated with C4BP for 1 h, or pseudo-typed particles that were not treated with C4BP. Post-infection, luciferase activity in the cells was measured using Pierce™ Firefly Luc One-Step Glow Assay Kit (Thermo Fisher) and CLARIOstar Plus Plate Reader (BMG Labtech).

### Statistical Analysis

GraphPad Prism 8.0 software was used to create all graphs. Two-way ANOVA was used to find the statistical significance of the data generated. Significant values were considered based on *p < 0.1, **p < 0.05, ***p < 0.01, and ****p < 0.001 between treated and untreated conditions. Error bars show the standard deviation among the samples.

## Results

### C4BP Directly Binds to Influenza A Virus in a Dose-Dependent Manner

**Figure 1:**
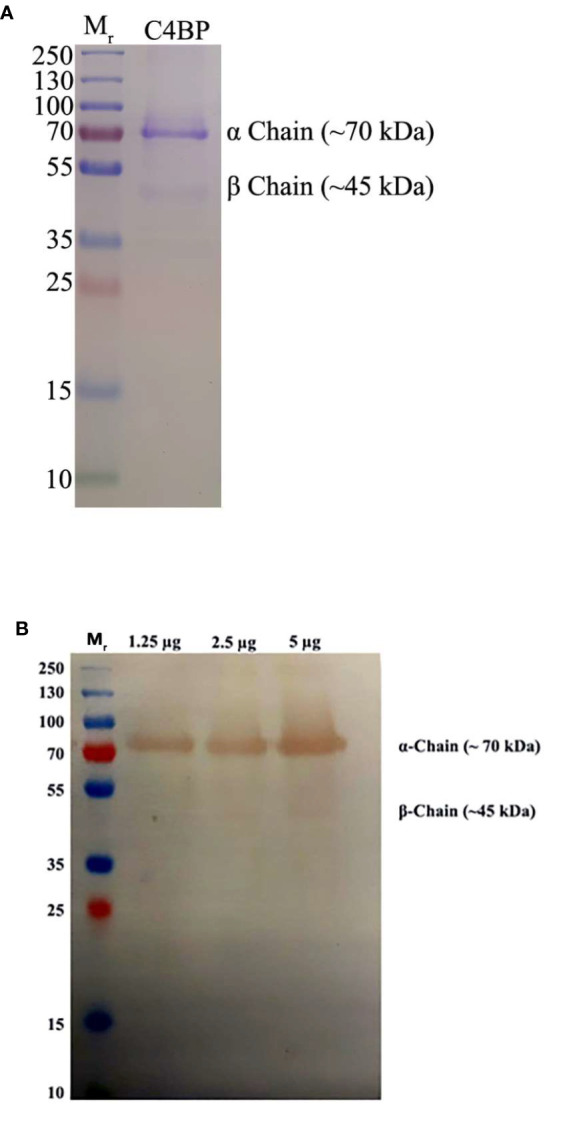
Characterization of purified C4BP. **(A)** SDS-PAGE (12% w/v acrylamide/bisacrylamide) was loaded with 5 µg of purified C4BP in lane 2, along with a protein ladder with a range of 250 to 10 kDa (Thermofisher) in lane 1. The denatured and reduced samples were run for 120 min at 90 V and stained with Coomassie brilliant blue to reveal protein bands corresponding to the α-chain (~70kDa) and a faint band corresponding to β-chain of C4BP (~45 kDa). **(B)** Immunoreactivity of the purified C4BP was analyzed by western blotting. A PVDF membrane with 1.25, 2.5, and 5 µg of purified C4BP in lanes 2, 3, and 4, respectively, along with a protein ladder with a range of 250 to 10 kDa (Thermofisher) in lane 1 was probed using rabbit-anti-human C4BP polyclonal antibodies (1:1,000) at room temperature for 1 h, followed by incubation with secondary goat anti-rabbit IgG HRP-conjugate (1:1,000) for 1 h at room temperature. Bands corresponding to α-chain (~70 kDa) and the β-chain (~45 kDa) of the C4BP were observed after developing the color using 3,3′-diaminobenzidine (DAB) substrate.

**Figure 2:**
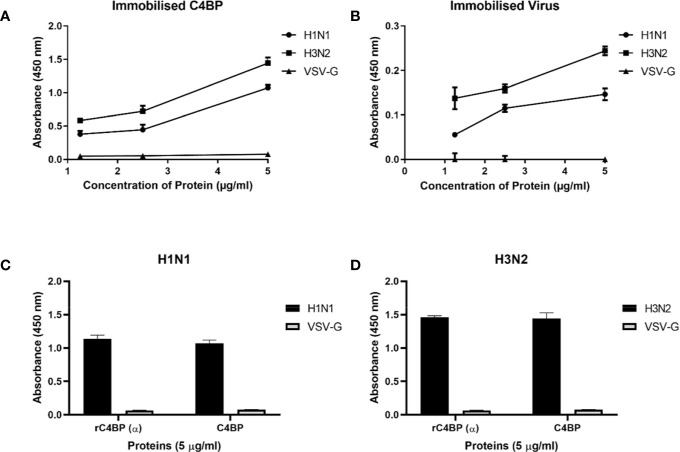
Binding interaction of immobilized C4BP to IAV subtypes **(A)**, and immobilized IAV subtypes to C4BP **(B)**. Microtiter wells were coated with a varied concentration of C4BP (5, 2.5, and 1.25 µg/ml) protein and incubated with constant volume of viruses (1,000 PFU/ml). Bound C4BP-IAV was probed with monoclonal anti-influenza virus H1 and polyclonal anti-influenza virus H3 antibody (1:5,000). Another ELISA was performed by coating a constant concentration of IAV subtypes (1,000 PFU/ml) and incubated with a varied concentration of C4BP protein (5, 2.5, and 1.25 µg/ml). IAV-C4BP interaction was detected using polyclonal rabbit anti-human C4BP antibodies and anti-rabbit IgG-HRP-conjugated antibody (1:5,000). A validation ELISA to show the detection of H1N1 **(C)** and H3N2 **(D)** binding to a recombinant C4BP (rC4BP) and plasma purified C4BP. Microtiter wells were coated with immobilized rC4BP and the plasma purified C4BP (5 µg/ml), and incubated with H1N1 or H3N2 (1,000 PFU/ml). The binding between immobilized proteins and IAV subtypes were detected using monoclonal anti-influenza virus H1 and polyclonal anti-influenza virus H3 antibody (1:5,000). VSV-G pseudo-typed lentiviral particles were used as a negative control; no significant binding was observed. The data were expressed as the mean of three independent experiments done in triplicates ± SEM.

Native C4BP, purified from plasma, was run on a 12% w/v reducing SDS-PAGE, which revealed a band corresponding to α-chain (~70 kDa), and one corresponding to β-chain (~45 kDa) (**Figure 1A**). The immunoreactivity of the protein was determined by performing a western blot with rabbit polyclonal anti-human C4BP antibodies, which yielded a band corresponding to the α-chain (~70 kDa) of C4BP. However, the β-chain (~45 kDa) was barely visible (**Figure 1B**). Indirect ELISA was then performed to examine the binding of C4BP to IAV subtypes. Binding of immobilized C4BP to the virus (**Figure 2A**), and conversely, of immobilized virus to C4BP (**Figure 2B**) was observed. As evident in **Figures 2A, B**, C4BP bound to H3N2 and H1N1 in a dose-dependent manner; binding to H3N2 was greater than to H1N1 (**Figure 2A**) subtype. Recombinant C4BP alpha chain (rC4BP) (**Supplementary Figure 1**) was also used to confirm its binding to the virus. No significant difference in binding efficiency was observed between the recombinant C4BP and the plasma purified C4BP (**Figures 2C, D**).

### C4BP Binds HA, NA, and M1 Proteins of Influenza A Virus

**Figure 3:**
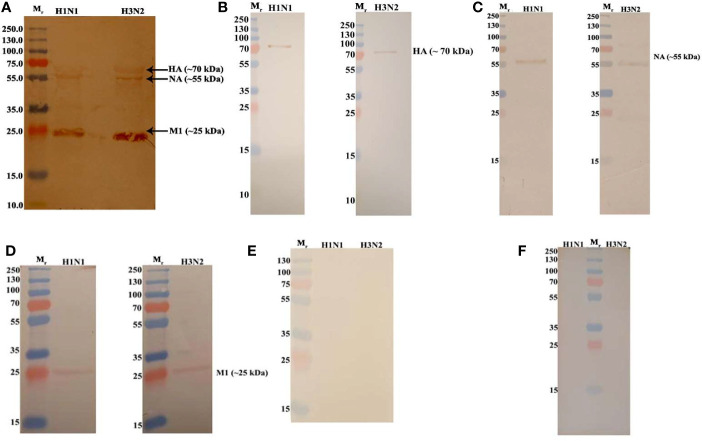
Far Western blot analysis showing C4BP binding to H1N1 and H3N2 proteins. PVDF membrane containing H1N1 and H3N2 virus proteins, separated by SDS-PAGE was blocked with 5% w/v skimmed milk and incubated with 5 µg/ml of C4BP **(A)** or without C4BP **(F)** followed by washing steps. The membrane was probed with rabbit-anti-human C4BP polyclonal antibodies (1:1,000) at room temperature for 1 h, followed by washing and incubation with secondary goat anti-rabbit IgG HRP-conjugate (1:1,000) for 1 h at room temperature. Bands corresponding to Hemagglutinin (HA) (~70 kDa), Neuraminidase (NA) (~55 kDa), and Matrix protein 1 (M1) (25 kDa) in the case of both H1N1 and H3N2 subtypes were revealed after developing colour using 3,3′-diaminobenzidine. The identities of C4BP bound IAV glycoproteins were confirmed using separate blots that were directly probed with anti-HA **(B)**, anti-NA **(C)**, anti-M1 **(D),** or anti-NS1 **(E)** antibodies.

In order to identify the IAV proteins that interact with C4BP, far-western blot analysis was performed. When SDS-PAGE separated IAV proteins were probed with C4BP, it revealed that C4BP interacted with IAV viral proteins Hemagglutinin(HA) (~70 kDa), Neuraminidase (NA) (~55 kDa) and Matrix protein 1 (M1) (~25kDa) (**Figure 3A**). The identities of C4BP bound IAV glycoproteins were confirmed using separate blots that were directly probed with anti-HA (**Figure 3B**), anti-NA (**Figure 3C**), anti-M1 (**Figure 3D**), or anti-NS1 antibodies (**Figure 3E**). IAV proteins separated by SDS-PAGE were probed directly with anti-C4BP antibodies to rule out non-specific interaction between the antibody and IAV proteins (**Figure 3F**). NS1 protein has similar molecular weight to M1, but the blot with antibodies confirmed M1 as the 25kDa species involved in binding.

### Influenza A Virus Binding Sites Within C4BP α-Chain Appear to Be Localized Within CCP4-5 and CCP7-8 Modules

**Figure 4:**
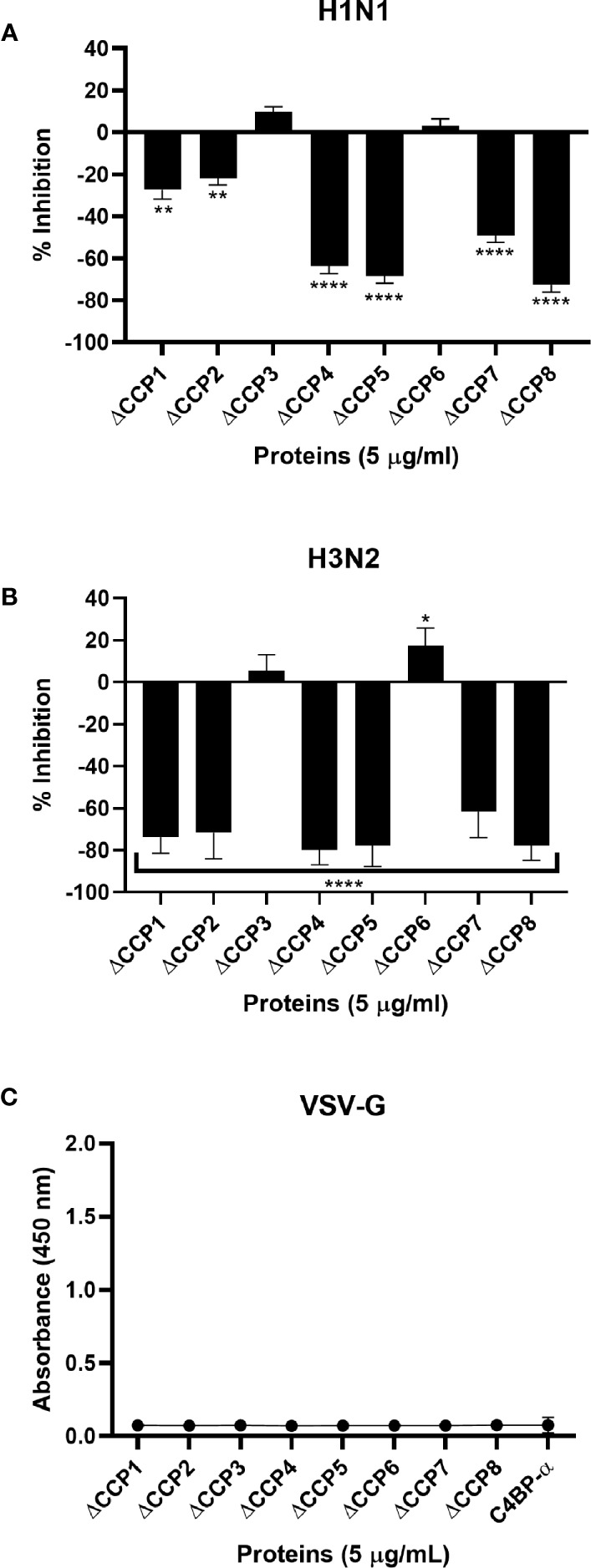
C4BP interacts with IAV through multiple CCP domains. ELISA showing binding of C4BP CCP deletion mutants to **(A)** H1N1, **(B)** H3N2 and **(C)** VSV-G: microtiter wells were coated with 5 µg/ml of the mutant C4BP protein (as described in **Table 1**). 1000 PFU/ml of H1N1 or H3N2 virus was added to all the wells. After incubation and washing steps, the bound virus was measured with either monoclonal anti-influenza virus H1, polyclonal anti-influenza virus H3 or polyclonal anti-VSV-G antibody. Results are normalised against full length C4BP α chain. The data were expressed as the mean of three independent experiments done in triplicates ± SEM. Significance was determined using the two-way ANOVA test (*p < 0.05, **p < 0.01, and ****p < 0.0001) (*n* = 3).

To identify the likely binding site on C4BP for the IAV, an indirect ELISA was performed using recombinant C4BP α-chain mutants lacking individual CCP domains. Recombinant C4BP lacking CCP4, 5, 7, or 8 showed relatively weaker binding to H1N1 and H3N2 subtypes. The deletion of CCP domain 8 reduced binding by approximately 75% for both subtypes when compared to full-length C4BP. Deletion of CCP7 resulted in reduced binding for H1N1 (~49%) as well as H3N2 (~62%). The CCP4 and 5 deletion mutants lost ~80% binding activity for H3N2 and ~60% for H1N1. Approximately 20% loss of binding for H1N1 was observed in the case of CCP1 and 2 deletants, but the same deletants showed ~80% reduction in binding to H3N2. The loss of CCP domains 3 and 6 caused a small increase in binding by approximately 10% for both IAV subtypes. This indicates that C4BP binds both IAV subtypes most likely involving CCPs 4-5 and 7-8 domains of its α-chain (**Figure 4**). However, in the case of the H3N2 subtype, and to a lesser extent H1N1 subtype, it appears that there is an additional binding interaction with CCPs 1-2 (**Figure 4B**). This may be consistent with greater binding to H3N2. VSV-G pseudo-typed lentivirus that was used as negative control showed no significant binding (**Figure 4C**).

### C4BP Inhibits H1N1 Replication

**Figure 5:**
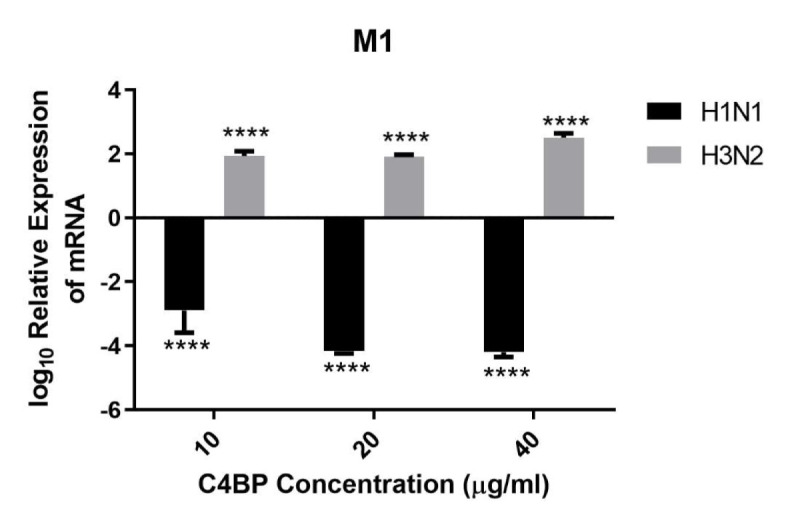
C4BP restricts replication of H1N1 and promotes replication of H3N2 in target human A549 cells. M1 expression of both H1N1 and H3N2 IAV after infection of A549 cells for 6 h was measured by qRT-PCR. A549 cells were challenged either with H1N1 or H3N2 (MOI 1) that were incubated with varying concentration of C4BP (10, 20, and 40 µg/ml). Cell pellets were harvested at 6 h to analyze the M1 expression of IAV. Cells were lysed, and RNA extracted was converted into cDNA. Infection was measured *via* qRT-PCR using M1 primers; 18S rRNA was used as an endogenous control. The relative expression (RQ) was calculated by using cells infected with respective viruses that were not treated with C4BP as the calibrator. The RQ value was calculated using the formula: RQ = 2^−ΔΔCt^. Assays were conducted in triplicates, and error bars represent ± SEM. Significance was determined using the two-way ANOVA test (****p < 0.0001) (*n* = 3).

HA, NA, and M1 are essential IAV proteins which have many functions from viral entry to virion budding, bringing about IAV infectivity and replication. Therefore, we investigated the effect of C4BP on cell infection. A549 cells were challenged with either H1N1 or H3N2 for 6 h that were previously incubated with C4BP. The efficacy of replication was estimated by comparing the M1 expression levels of the virions treated with C4BP (10, 20, or 40 µg/ml) to the cells infected with IAV but, not treated with C4BP. M1 mRNA levels were downregulated by C4BP in the case of H1N1 (−4 log_10_), suggesting that the interaction of C4BP with H1N1 reduced the replication efficiency of the virus at an early stage of the replication cycle. However, in the case of A549 cells challenged with H3N2, M1 mRNA levels were found to be upregulated (2 log_10_), suggesting an increase in IAV replication efficacy. Taken together, these results suggest that C4BP differentially modulates viral entry, and thus, replication efficacy among the IAV subtypes (**Figure 5**).

### C4BP Is an Entry Inhibitor for H1N1

**Figure 6:**
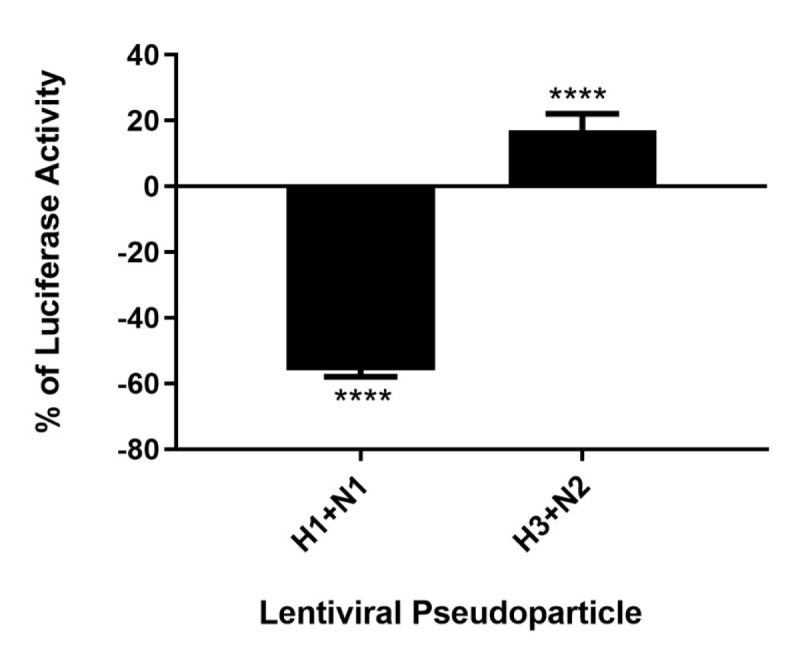
C4BP modulates viral entry in a strain-dependent manner. Luciferase reporter activity of C4BP treated MDCK cells infected with matched H1+N1 and unmatched H3+N2 lentiviral pseudo-particles. H1N1 particles exhibited a 55% decrease in viral entry compared to controls, while a 17% increase in viral entry in case of H3N2 was observed. Results are normalized against cells infected with the particles not treated with C4BP. Significance was determined using the unpaired one-way ANOVA test (****p < 0.0001) (*n* = 3).

As C4BP was found to interact with HA and NA, the IAV proteins that play an integral role in viral host cell recognition and entry, lentiviral pseudo-particles were used to study the entry process independent of other IAV components. The pseudo-particles expressing the respective IAV coat proteins with an HIV-Luciferase backbone were used as a safer alternative to assess the role of C4BP as an entry modulator of H1N1 and H3N2. This system contains a single packaging plasmid (psPAX2) encoding genes including Gag, Pol, and Tat. pHIV-Luciferase was used as a lentiviral transfer plasmid, which is flanked by long terminal repeat (LTR) sequences and designed to express the firefly luciferase reporter. Thus, pHIV-Luciferase is replication-incompetent which contains an additional sequence deletion in the 3′ LTR leading to viral self-inactivation post-integration. Purified H1+N1 and H3+N2 pseudo-typed particles pre-incubated with C4BP (20 μg) were used to infect MDCK cells. The lentiviral particles not treated with C4BP were used as controls. A ~56% reduction in luciferase reporter activity was observed when MDCK cells infected with C4BP-treated H1+N1 pseudo-typed particles were compared to their untreated counterparts. However, an opposite effect, although not so dramatic, was observed following C4BP treatment in the case of H3N2 unmatched pseudo-particles. A ~17% increase in luciferase activity was observed in the treated samples when compared to the control (**Figure 6**). C4BP protein seems to be acting as an entry inhibitor for H1N1, but a weak facilitator of infection by H3N2.

### C4BP Modulates Pro-inflammatory Cytokine/Chemokine Response in IAV-Challenged A549 Cells

**Figure 7:**
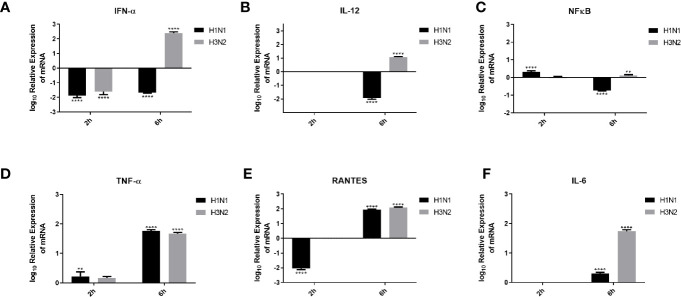
Cytokine modulation by C4BP of IAV infected A549 cells. H1N1 or H3N2 incubated with 10µg/ml of C4BP was used to infect A549 cells. Cell pellets were harvested at 2 h and 6 h to analyse the expression of cytokines and chemokines. Cells were lysed, and purified RNA was converted into cDNA. The expression levels of cytokines IFN-α **(A)**, IL-12 **(B)**, NF-κB **(C)**, TNF-α **(D)**, IL-6 **(F)** and chemokine RANTES **(E)** were measured using qRT-PCR, and the data were normalised *via* 18S rRNA expression as a control. The relative expression (RQ) was calculated by using cells infected with respective viruses not treated with C4BP as the calibrator. The RQ value was calculated using the formula: RQ = 2^−ΔΔCt^. Assays were conducted in triplicates, and error bars represent ± SEM. Significance was determined using the two-way ANOVA test (**p < 0.01, and ****p < 0.0001) (*n* = 3).

Pro-inflammatory cytokines and chemokines, such as IL-1, IL-6, IL-12, IFN-α, and NF-κB transcription factor, are a hallmark of IAV infection (34). In order to understand the effect of C4BP on the pro-inflammatory cytokines produced during IAV infection, A549 cells were challenged with H1N1 and H3N2 viruses (MOI 1) preincubated with C4BP. qRT-PCR was then carried out using total RNA from the cells (**Figure 7**), using cells infected with IAV that were not treated with C4BP as the control. C4BP treated H1N1 challenged A549 cells showed lower levels of IFN-α (-2 log_10_) at both 2 and 6 h, whereas cells challenged with C4BP treated H3N2 exhibited higher levels of IFN-α 6 h post-infection (**Figure 7A**). IL-12 is essential for the generation of cell-mediated immunity *via* the production of IFN-γ by helper type 1 T (Th1) cells, which leads to the elimination of the virus (35). Levels of IL-12 at 2 h in case of A549 cells challenged with either C4BP treated H1N1 or H3N2 were not detectable (**Figure 7B**). At 6 h in C4BP treated H1N1 challenged cells, IL-12 levels were downregulated (−2 log_10_), while in cells challenged with C4BP treated H3N2 the levels were upregulated (1 log_10_) (**Figure 7B**). As interferon response is triggered by the detection of viral components within infected cells, and IL-12 induction requires live viruses, these results are consistent with the previously observed levels of M1 transcript.

The transcription factor, NF-κB, is a regulator of antiviral cytokines such as IFN-β, which initiates a strong type I IFN immune response. IAV induces IFN expression *via* NF-κB as viral titer levels are higher in cells treated with an anti-IFN-α/β receptor antibody and not in dominant-negative IκBα expressing cells (36). NF-κB mRNA levels at 2 h were slightly upregulated in C4BP treated H1N1 challenged cells and no significant change in its levels were observed in cells challenged with C4BP treated H3N2 (**Figure 7C**). Cells infected with C4BP treated H1N1 at 6 h exhibited lower levels of NF-κB (−1 log_10_), and no significant changes were observed in C4BP treated H3N2 infected cells (**Figure 7C**). The lower levels of NF-κB can be attributed to either low viral titer in the case of H1N1 or the IAV non-structural protein NS1 which acts as a major suppressor of NF-κB activation (37).

Increased levels of IL-6, TNF-α, and IFN-γ, have been detected in individuals infected with acquired acute as well as severe seasonal IAV-infected patients (38, 39). Production of TNF-α during IAV infection correlates with morbidity and mortality rates in IAV infected humans and macaques (40, 41). Six hours post-infection, TNF-α mRNA levels were upregulated (2 log_10_) in A549 cells infected with C4BP treated H1N1 or H3N2, while at the earlier 2-h time point a slight upregulation was observed (**Figure 7D**).

RANTES is a C-C chemokine and a potent chemoattractant for monocytes, T lymphocytes, basophils, and eosinophils. IAV, *via* its HA, can induce RANTES expression, probably through the activation of NF-κB, and cause airway hypersensitivity by inducing the continuous infiltration of eosinophils (42). C4BP treated H1N1 challenged A549 cells exhibited lower levels of RANTES expression (−2 log_10_) at 2 h compared to the control; no changes were observed in cells challenged with C4BP treated H3N2 (**Figure 7E**). RANTES expression (2 log_10_) was upregulated at 6 h in the case of both subtypes (**Figure 7E**). Enhanced levels of IL-6 have been observed in the serum and lungs of IAV infected patients. It is unknown whether IL-6 in IAV infected patients contributes to lung pathology caused by the viral infection or if increased levels of IL-6 contribute to a protective mechanism against IAV (39, 43). Levels of IL-6 at 2 h in C4BP treated A549 cells challenged with either virus were not detectable (**Figure 7F**). At 6 h in C4BP treated H3N2 infected cells, IL-6 levels (2 log_10_) were upregulated; in cells challenged C4BP treated H1N1 the IL-6 levels (0.5 log_10_) were also upregulated (**Figure 7F**). Studies also suggest that signals mediated by IL-6 are crucial for survival against a non-lethal dose of H1N1 infection, and IL-6/IL-6R deletion inhibits H1N1 clearance together with reduced neutrophils levels in the lungs of infected mice (44).

## Discussion

The Influenza virus, an upper respiratory tract pathogen, is known to have caused some of the deadliest pandemics in history. To develop better IAV treatment strategies and mitigate the risk of the current viral strains evolving to a more pathogenic one by reassorting among themselves, a better understanding of the immune mechanisms against IAV is required. Here, we examined the complement-independent role of C4BP, a complement regulator, against IAV. Many pathogens such as adenoviruses, *Yersinia pestis*, and *Aspergillus* spp. are known to bind C4BP to promote survival and proliferation (15). C4BP is a major fluid phase inhibitor of the classical and the lectin pathways, making it a prime target for the exploitation by pathogens to circumvent the host’s potent humoral immune system, such as complement. In this study, we sought to understand the interactions between C4BP and IAV in a serum-free system and explore any complement-independent functions of C4BP with respect to IAV.

We first established protein-protein interactions between C4BP and IAV subtypes. C4BP α chain was found to bind IAV subtypes (**Figures 2A, B**), and H3N2 was found to bind C4BP with higher affinty than H1N1. This interaction with the α chain of C4BP was further confirmed using rC4BP (**Figures 2C, D**). The C4BP α chain was found to interact with viral envelope protein HA, NA, and surprisingly, M1 (**Figure 3**). As the M1 protein lies beneath the lipid layer, the interaction likely occurs by the abundantly present non-packaged M1 that are released from ravaged cells during late stages of infection to help increase the odds of survival of the virus (45).

It was also possible that C4BP interacted with IAV NS1, but the signal was masked by the interaction of C4BP with M1 due to the low concentrations of NS1 when compared to M1, as proteins share similar molecular weights. Avirutnan *et al.* have shown that Flavivirus NS1 restricted the activation of classical and lectin pathways by binding to C4BP, thereby enabling the virus to evade C4b mediated lysis (5). Since both NS1 proteins share independent immunoregulatory functions, the binding of Influenza NS1 with C4BP was also considered. Far western blot analysis and direct western blot analysis confirmed that it was M1 that C4BP bound to at ~26 kDa; probing with monoclonal Anti-IAV NS1did not reveal any bands corresponding to NS1 protein. Furthermore, multiple sequence alignment and identity-similarity analysis on IAV NS1 [(Accession #: P03495; P03496) and Flavivirus NS1 (Accession #: Q6J3P1 Residues: 779-1130) (P29991) Residues: 776-1127; P06935 (Residues: 788-1139)] performed using Clustal Omega and Sequence Manipulation Suite (Data not shown) revealed IAV NS1 and Flavivirus NS1 have sequence similarity scores that are lower than 25%.

Use of deletion mutants lacking specific CCP domains of C4BP revealed that H1N1 and H3N2 subtypes interacted *via* multiple CCP domains, CCP1-2, CCP4-5, and CCP7-8 of the C4BP α-chain. It is known that the Flavivirus NS1-C4BP binding is mediated through CCP2-5 and 8. Since CCP8 is near to the C-terminal oligomerization domain, its loss could affect the conformational structure of C4BP, which can affect the accessibility of NS1 to CCP2-5 (5). Similarly, the deletion of CCP8 reduced IAV-C4BP binding by ~75% (**Figure 4**). Deletion of CCP4-5 inhibited binding by ~79% to H3N2 and ~67% to H1N1 (**Figure 4**). C4BP binds C4b through CCP1-3, with domains 2 and 3 being the most critical for the interaction (10). The loss of CCP1-2 reduced binding efficiency of H3N2 and, to a lesser extent, of H1N1 to C4BP (~80% for H3N2 and ~20% for H1N1) (**Figure 4**). It is possible that the IAV virus, like other pathogens such as *S. pyogenes*, *Neisseria gonorrhoeae*, *Bordetella pertussis*, *Moraxella catarrhalis*, *Borrelia recurrentis*, *Candida albicans*, and *Hemophilus influenzae,* is exploiting the natural binding site for C4b (8). This preference for the site can be attributed to its immutability, accessibility and the fact that the region may contain intrinsic properties which favor ligand binding (46). Furthermore, this increased binding affinity between CCP1-2 and H3N2 may explain the difference in the binding efficiency of C4BP to H3N2 when compared to H1N1, as seen in **Figure 2**. As plasma purified C4BP is a glycoprotein, and therefore, is likely to contain N-glycosides that are sialylated; it is possible for sialic acid residues to bind to HA/NA. C4BP [UniProtKB - P04003 (C4BPA_HUMAN)] has N-linked glycosylation only in CCP3 and CCP8 and so sialic acid is likely to occur only at these sites. Our results do not implicate CCP3 in binding, but it remains a possibility that CCP8 binding could involve sialic acid.

It is likely that C4BP is regulating viral replication levels by modulating viral entry. To establish this, second-generation matched and unmatched lentiviral vectors, pseudo-typed for H1+N1 and H3+N2, respectively, were generated. This system was selected as a safe alternative method to mimic the structure and surfaces of IAV and prove that C4BP acts as an entry inhibitor in cells transduced with pseudo-typed IAV particles that are restricted to only one replicative cycle. A luciferase reporter assay revealed a 56% reduction in H1N1 pseudo-typed viruses treated with C4BP that entered the cells, corresponding with the 4-fold decrease observed in the M1 mRNA levels of H1N1 infected cells (**Figures 5** and **6**), while a slight increase (~17%) was observed in case of the H3N2 pseudo-particles (**Figure 6**), consistent with M1 levels. This suggests that C4BP acts as an entry inhibitor for H1N1 while it has limited restrictive potential against H3N2.

Recently, factor H, the principal regulator of the alternative pathway, and vaccinia virus complement control protein (VCP), a homologue of factor H, were found to interact with IAV in a manner similar to C4BP (32). MDCK cells infected with H1+N1pseudo-typed lentiviral particles resulted in approximately 25% and 45% reduction in luciferase activity after treatment with factor H or VCP, respectively. However, a 50% increase in luciferase activity in MDCK infected with H3+N2 pseudo-typed lentiviral particles was observed when treated with factor H, and 30% when treated with VCP was observed. Hence, the treatment with these proteins resulted in restriction of IAV entry and replication by 4-fold for the H1N1 subtype, while promoting entry and replication by 2-fold for H3N2. Thus, using lentiviral vectors, pseudo-typed for H1+N1 and H3+N2, Murugaiah et al. demonstrated the ability of Factor H and VCP to modulate IAV entry, in a subtype dependent manner, similar to C4BP (32). As seen in the case of C4BP, Murugaiah et al. also suggested that FH and VCP to accomplished the subtype dependent entry modulation via interaction with HA, NA, and M1. Additionally, FH and VCP were also shown to trigger an anti-inflammatory response in case of H1N1 subtype while leading to pro inflammatory response in H3N2 subtype. The inflammatory response was also modulated in a subtype-specific manner. Transcriptional levels of IFN-α, TNF-α, IL-12, IL-6, and IFN-α were upregulated, while RANTES was downregulated following factor H treatment for the H1N1 subtype at 6 h post-infection. In the case of the H3N2 subtype, mRNA levels of these pro-inflammatory cytokines were enhanced (32). The findings of this study highlight a possible common mechanism that is used by C4BP, VCP, and Factor H that contain CCP domains in modulating the entry of IAV subtypes. The heparin-binding sites on Factor H (surface of CCP7, CCP13 and CCP20) and VCP (tip of module 4) are also found on C4BP α chain (CCP domains 1-2) (9, 47). These sites may provide a possible explanation for the effect observed on viral entry modulation among the IAV subtypes. In this study, we have shown that IAV subtypes bind C4BP *via* the heparin-binding site containing CCP domain 1-2 (**Figure 4**). We suspect the overlapping heparin-binding and IAV binding sites may contribute to the variation observed between the subtypes. C4BP has been shown to enhance adenovirus uptake in hepatocytes by providing a bridge between the adenovirus fiber knob domain and cell surface Heparan Sulfate Proteoglycans (HSP) (16); we speculate that the increased efficiency observed between the binding of C4BP’s CCP1-2 domain and H3N2 subtype compared to H1N1 subtype may contribute to the increased H3N2 virion entry in a similar fashion. The similarity of the results between the interaction of C4BP, Factor H, and VCP to IAV subtypes further support this idea and requires further study by treating cells with heparinase prior to C4BP treatment and infection to confirm whether the variation between the subtypes may be modulated by the interaction with HSP.

Another explanation for differences in outcome in spite of C4BP binding both viruses may arise from the local variations found between H1 and H3 or N1 and N2 or the variation in C4BP binding sites to HA or NA. HA and NA are classified into two groups regardless of their serotype identification. In case of NA, N1 belongs to group 1, which lacks an additional cavity next to the active site that is created by the movement of “150 loop” during conformational changes caused by the binding of substrate to the active site (48). N2 belongs to group 2 which lacks the “150 loop”, and hence, the additional cavity (48). NA functions in virus internalization by enabling movement of the virus across the cell surface from an endocytosis inactive site on the cell to an active site, thereby increasing the efficiency of viral uptake (49). NA has also been shown to boost HA-dependent influenza virus fusion and infectivity by employing a cell-cell fusion assay and an HIV-based pseudo-typed infectivity assay (50). It is possible that the structural differences between N1 and N2 after binding to C4BP may affect their role in virus internalization. Similarly, HA can also be classified into two groups based on structural characteristics, with H1 in group 1 and H2 in group 2 (51). Hence, localized variations found on HA could influence potential binding pocket for C4BP on the HA, as seen in the case of Stachyflin (52). These structural variations in HA and NA may also explain the increased binding observed between C4BP and H3N2. It will be important to investigate the effect of differential interplay between HA and NA of H1N1 and H3N2 viruses on the infection modulation activity of C4BP. Additionally, the variation in binding efficiency of CCP1-2 between H3N2 and H1N1 suggests that the binding while overlapping, does not occur at identical sites, as seen in the case of C4BP binding to C4b, *S. pyogenes*, or *B. pertussis* (46, 53, 54). It may be possible that this variation and difference in binding efficiency further affects downstream interactions such as C4BP or IAV’s interaction with other receptors or proteins that may affect viral entry (55, 56).

During an IAV infection, elevated levels of cytokines and chemokines are observed, including IL-6, TNF-α, IFNs, IL-1β, RANTES, IL-8, MIP1β, and MCP1 (57–60). Inflammation is a critical part of the immune response against IAV infection. Cytokines such as TNF-α, IL-12, and IFNs are known to play a significant role to cellular inflammation. To study the modulation of these cytokines by C4BP, we performed qPCR on A549 cells infected with C4BP treated H1N1 or H3N2 (treated) and standardized them against A549 cell infected under similar conditions using viruses that were not treated with C4BP (untreated) to obtain the relative expression levels of the cytokines. Type I interferons like IFN α and IFN β are critical mediators of virus clearance during an IAV infection. Six hours post-infection in the treated cells, IFN-α mRNA levels were downregulated in case of H1N1 (−2 log_10_) and upregulated (2.5 log_10_) in case of C4BP treated H3N2 infected cells (**Figure 7A**). This suggests the formation of an antiviral state in the infected and its neighbouring cells, thereby reducing viral replication and disease severity (61, 62). The elevated levels of IFN-α mRNA observed in A549 cells challenged with the C4BP treated H3N2 subtype suggest that C4BP is being protective by promoting a type 1 interferon response despite not acting as an entry inhibitor for H3N2. The cytokine mRNA expression was downregulated (−2 log_10_) in the case of both viruses 2 h post-infection and can be attributed to the low viral load present at the time (**Figure 7A**).

We found that IL-12 mRNA levels were too low to be detected 2 h post-infection, but the relative mRNA upregulation (1 log_10_) was observed 6 h post-infection in cells infected with C4BP treated H3N2 compared to its control (**Figure 7B**). This clearly indicates the presence of a response that would culminate in the prevention of viral replication, activation of cytotoxic T lymphocytes and early NK cell-dependent gamma interferon production (63). The entry inhibitory effect of C4BP on H1N1 subtype, and the subsequent lower viral replication and load is further demonstrated by the downregulation (−2 log_10_) of IL-12 mRNA in the C4BP treated H1N1-infected cells compared to their untreated counterparts 6 h post-infection (**Figure 7B**).

The TNF-α–NF-κB axis leads to the expression of a number of inflammatory genes, induction of apoptosis and production of ROS (64–66). NF-κB mRNA in cells infected with C4BP treated H1N1, 2 h post-infection was found to be relatively upregulated (~0.3 log_10_), while a downregulation (~−0.7 log_10_) of the mRNA was recorded 6 h post-infection when compared to the untreated samples (**Figure 7C**). In case of cells infected with C4BP treated H3N2, we found an upregulation of NF-κB mRNA 6 h post-infection, while no significant modulation was observed 2 h post-infection (**Figure 7C**). This variation in NF-κB mRNA levels 6 h post-infection among the IAV subtype further confirms the subtype dependent entry inhibitory action of C4BP on IAV. When compared to their untreated counterparts, the cells infected with either C4BP treated H1N1 or H3N2 were found to upregulate TNF-α mRNA (**Figure 7D**), indicating a robust inflammatory response at both 2 h (0.2 log_10_) and 6 h (~1.7 log_10_) post-infection (67, 68).

RANTES is an important chemokine that been associated with extensive inflammation of the lung in cases of avian Influenza and increased mortality in IAV challenged CCR5^−/−^ mice (69). RANTES mRNA expression levels in treated H1N1 challenged cells 2 h post-infection, was found to be significantly downregulated (−2 log_10_) while it was not detected in case of H3N2 (**Figure 7E**). RANTES mRNA levels in cells were upregulated observed in case of both C4BP treated viral subtypes (2 log_10_) 6 h post-infection, suggesting an active recruitment of leukocytes to the inflammatory sites (**Figure 7E**).

Another cytokine associated with both pro-inflammatory and anti-inflammatory effects is IL-6 (70). Its synthesis is upregulated during infection to simulate the adaptive immune response by inducing differentiation of activated B cells and CD4^+^ T cells (71). In the case of IAV infection, IL-6 seemed to have a protective role in murine models, promoting viral clearance and limiting inflammation (72). IL-6 mRNA levels at 6 h was found to be upregulated in case of H1N1 (0.5 log_10_) and H3N2 (1.5 log_10_) (**Figure 7F**) after C4BP treatment. These results suggest that C4BP may entice differential adaptive immune response.

It would be interesting to study if C4BP can also bind avian IAV. Another area to explore further would be investigate in humanized murine models to understand better the impact of C4BP-IAV interaction in the microenvironment of the respiratory system, and elucidation of mechanism resulting in the subtype-dependent entry modulation of IAV by C4BP.

In conclusion, we show that C4BP differentially regulates the efficacy of Influenza A virus replication in a subtype-dependent manner by modulating their entry into the cells, and provide an interesting candidate to be developed into a HA and NA-based inhibitor against IAV infections. Recombinant forms of CCP4+5 and/or CCP7+8 are likely to be excellent vehicles for therapeutic development against IAV pandemic H1N1 subtype.

## Data Availability Statement

The raw data supporting the conclusions of this article will be made available by the authors, without undue reservation.

## Author Contributions

PV, VM, and NB carried out most of the experiments. NT, HK, SA, and MA-A provided crucial reagents. FA-M, BN, RS, and UK managed the experimental details and analyzed the data. PV and VM wrote the first draft. UK, RS, and BN edited the manuscript. All authors contributed to the article and approved the submitted version.

## Funding

The authors acknowledge the International Scientific Partnership Programme (ISPP) at King Saud University, Riyadh, for funding *via* ISPP-145.

## Conflict of Interest

The authors declare that the research was conducted in the absence of any commercial or financial relationships that could be construed as a potential conflict of interest.

The reviewer KR declared a shared affiliation with one of the authors, RS, to the handling editor at the time of review.

## Supplementary Material

Supplementary Figure 1
**(A)** SDS-PAGE (12% w/v acrylamide/bisacrylamide) was loaded with 5 µg of recombinant C4BP α chain in lane 2, along with a protein ladder with a range of 250 kDa to 10 kDa (Thermofisher) in lane 1. The denatured and reduced samples were run for 120 minutes at 90V and stained with Coomassie brilliant blue to reveal protein bands corresponding to the α-chain (~70kDa). **(B)** Immunoreactivity of the recombinant C4BP α chain was analysed by western blotting using rabbit-anti-human C4BP polyclonal antibodies (1:1000) at room temperature for 1 h, followed by incubation with secondary goat anti-rabbit IgG HRP-conjugate (1:1,000) for 1 h at room temperature. Bands corresponding to α-chain (~70kDa) of the C4BP were observed after developing the colour using 3,3′-diaminobenzidine (DAB) substrate.
