# Supplementary figures and images for: C4b Binding Protein Acts as an Innate Immune Effector Against Influenza A Virus
Source: Front Immunol. 2021 Jan 8;11:585361. doi: 10.3389/fimmu.2020.585361 (PMC7820937; doi:10.3389/fimmu.2020.585361)

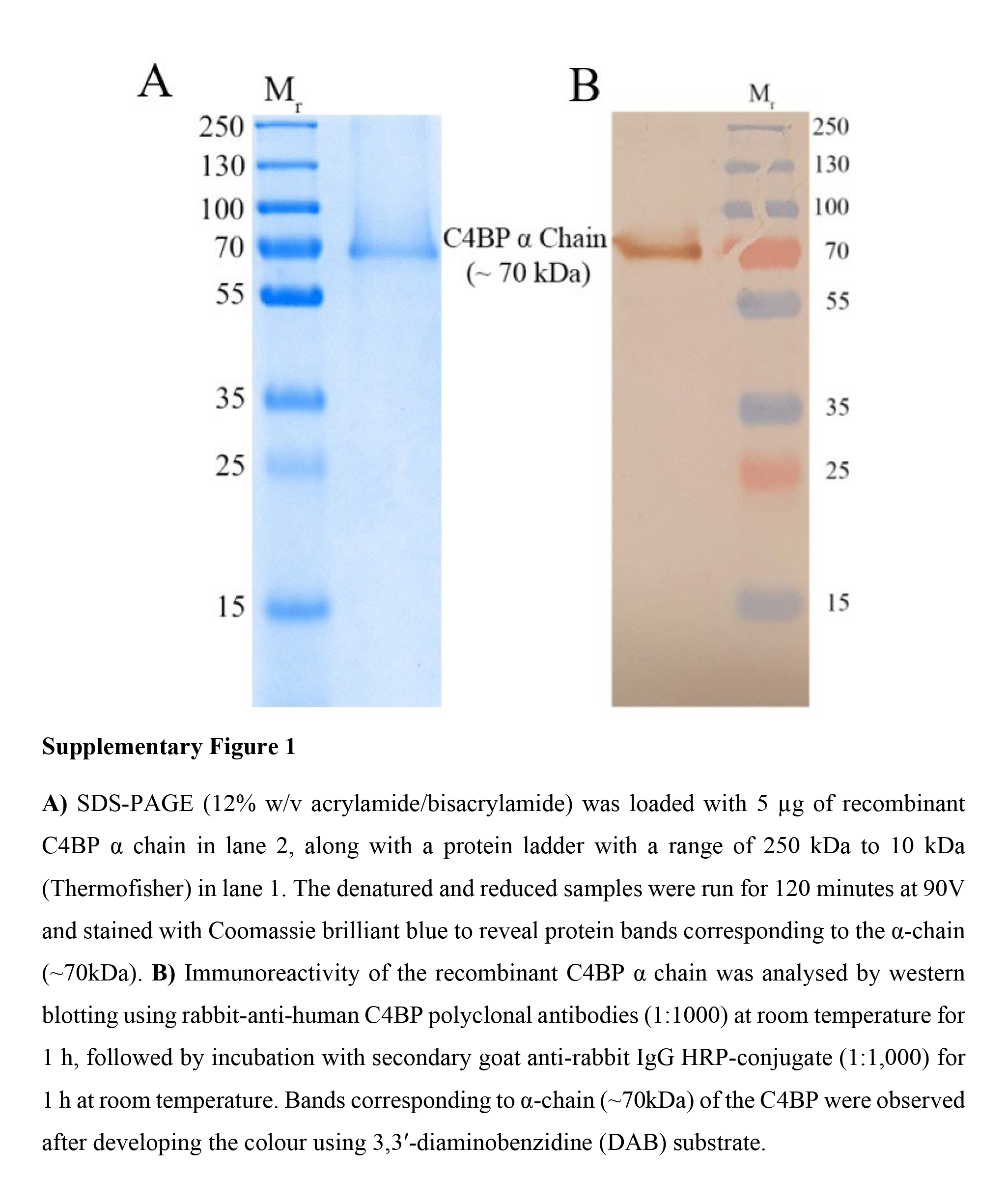

Supplement: Supplementary Figure 1 — (A) SDS-PAGE (12% w/v acrylamide/bisacrylamide) was loaded with 5 µg of recombinant C4BP α chain in lane 2, along with a protein ladder with a range of 250 kDa to 10 kDa (Thermofisher) in lane 1. The denatured and reduced samples were run for 120 minutes at 90V and stained with Coomassie brilliant blue to reveal protein bands corresponding to the α-chain (~70kDa). (B) Immunoreactivity of the recombinant C4BP α chain was analysed by western blotting using rabbit-anti-human C4BP polyclonal antibodies (1:1000) at room temperature for 1 h, followed by incubation with secondary goat anti-rabbit IgG HRP-conjugate (1:1,000) for 1 h at room temperature. Bands corresponding to α-chain (~70kDa) of the C4BP were observed after developing the colour using 3,3′-diaminobenzidine (DAB) substrate. [file Image_1.tif]
